# Effects of information on acceptance of the final disposal of soils removed after the Fukushima accident and negative emotional responses to it

**DOI:** 10.1093/jrr/rrag035

**Published:** 2026-05-18

**Authors:** Michio Murakami, Momo Takada, Yukihide Shibata, Kosuke Shirai, Susumu Ohnuma, Tetsuo Yasutaka

**Affiliations:** Center for Infectious Disease Education and Research, The University of Osaka, 1-10, Yamadaoka, Suita, Osaka 565-0871, Japan; EIPM Center, The University of Osaka, 1-10, Yamadaoka, Suita, Osaka 565-0871, Japan; Integrated Research Center for Nature Positive Technology, National Institute of Advanced Industrial Science and Technology, 1-1-1 Higashi, Tsukuba, Ibaraki 305-8567, Japan; Research Institute for Geo-Resources and Environment, Geological Survey of Japan, National Institute of Advanced Industrial Science and Technology, 1-1-1 Higashi, Tsukuba, Ibaraki 305-8567, Japan; Department of Behavioral Science, Faculty of Humanities and Human Sciences, Hokkaido University, Kita 10 Nishi 7, Kita-ku, Sapporo, Hokkaido 060-0810, Japan; Research Institute for Geo-Resources and Environment, Geological Survey of Japan, National Institute of Advanced Industrial Science and Technology, 1-1-1 Higashi, Tsukuba, Ibaraki 305-8567, Japan; Department of Behavioral Science, Faculty of Humanities and Human Sciences, Hokkaido University, Kita 10 Nishi 7, Kita-ku, Sapporo, Hokkaido 060-0810, Japan; Integrated Research Center for Nature Positive Technology, National Institute of Advanced Industrial Science and Technology, 1-1-1 Higashi, Tsukuba, Ibaraki 305-8567, Japan; Research Institute for Geo-Resources and Environment, Geological Survey of Japan, National Institute of Advanced Industrial Science and Technology, 1-1-1 Higashi, Tsukuba, Ibaraki 305-8567, Japan

To the editor:

Approximately 13 million m^3^ of soils and incineration ash removed after the 2011 Fukushima accident are stored in interim facilities near the plant. Although the law stipulates that the necessary measures shall be taken to complete the final disposal of these soils and other materials outside Fukushima Prefecture by 2045, the disposal site has not yet been determined. Previous studies show that public acceptance is associated with socio-psychological factors such as dread risk perception (belief that acceptance elicits intuitive dread), social benefits (belief that acceptance benefits Japan), trust in authorities, intergenerational expectations (perceived expectations from past and future generations as well as surrounding others regarding acceptance), inequity (belief that acceptance only in one’s region is unfair), and protected values (belief that acceptance should never be permitted for any reason whatsoever) [1–3]. To promote final disposal, effective information strategies must be identified; however, evidence remains limited.

We focused on perceived inequity, intergenerational expectations and social benefits, which may be modifiable through information, and examined their effects on acceptance. We anticipated that perceived inequity could be alleviated by emphasizing the burden placed on Fukushima residents. Similarly, intergenerational expectations would be increased by emphasizing comparisons with others. Social benefits would be enhanced by explaining that acceptance of final disposal would contribute to the greater good of Japan as a whole. Negative emotional responses to the information were also assessed, as it is important to ensure that the presentation of such information does not itself induce adverse reactions from the perspective of avoiding coercion in information presentation. Four information types were used: A (control): soils are stored temporarily and will be disposed of outside Fukushima by 2045; B: A plus the excessive burden already borne by Fukushima residents; C: A plus that 60% of people would accept disposal near their homes if local opinions were reflected [4]; D: A plus that acceptance contributes to Japan’s overall development. The hypotheses were: (i) Acceptance of B–D exceeds A; (ii) negative emotions do not differ among conditions; (iii) Compared with A, B reduces inequity; C increases intergenerational expectations; and D increases social benefits.

This study was approved by the Ethics Committee of the Center for Infectious Disease Education and Research at the University of Osaka (2023CRER1225; 2025CRER0513–2) and pre-registered (https://doi.org/10.17605/OSF.IO/DHMUK). An online survey (January 29–February 1, 2024) targeted Japanese residents aged 20–69 (excluding Fukushima) through Cross Marketing. Participants (*n* = 4000; 1000 per group) were quota-sampled by age and gender. Sample size was based on ~670 participants per group for 99% confidence and 5% margin of error [5]. After consent, completion of instructional manipulation checks (IMCs) [6], and questionnaires on demographics, respondents received an explanation of final disposal and comprehension checks. They were then randomly assigned to one condition (A–D) and rated acceptance of disposal near their residence (4-point scale) and negative emotional responses to the information (involuntariness [reverse], forced nature, distress and need for improved explanation; 5-point scale) [7]. Inequity, intergenerational expectations, social benefits, protected values and dread risk perception regarding health effects associated with acceptance were also measured. Details of these items were described elsewhere [1, 3, 4]. As pre-registered, group differences were analysed using Kruskal–Wallis tests with Dunn–Bonferroni post hoc comparisons or chi-square tests. Dread risk perception was also analysed across groups, although this analysis was not pre-registered, because of its potential association with acceptance. Planned subgroup analyses by protected values were not conducted because protected values differed significantly across groups (*P* < 0.05).

Of 16 355 respondents, exclusions (non-consent, IMC failures, ineligible age, gender, residence, survey company criteria, dropout or content-check failures) yielded 4000 participants representative of Japan’s age and gender distribution (Fig.  1). No significant demographic differences were observed among groups (Table 1). Mean acceptance was higher in B and D, with significant overall group differences but no significant post hoc contrasts. Significant differences were found in involuntariness, forced nature and need for improvement. C showed the lowest involuntariness; B and D were also lower than A. Forced nature was lower in C than in A, and B showed less need for improvement than A. Intergenerational expectations, protected values and dread risk perception differed significantly across groups. B showed higher intergenerational expectations than A and lower protected values than A and C. B and D showed lower dread risk perception than C. Effect sizes exceeded 0.1 only for involuntariness.

**Fig. 1:**
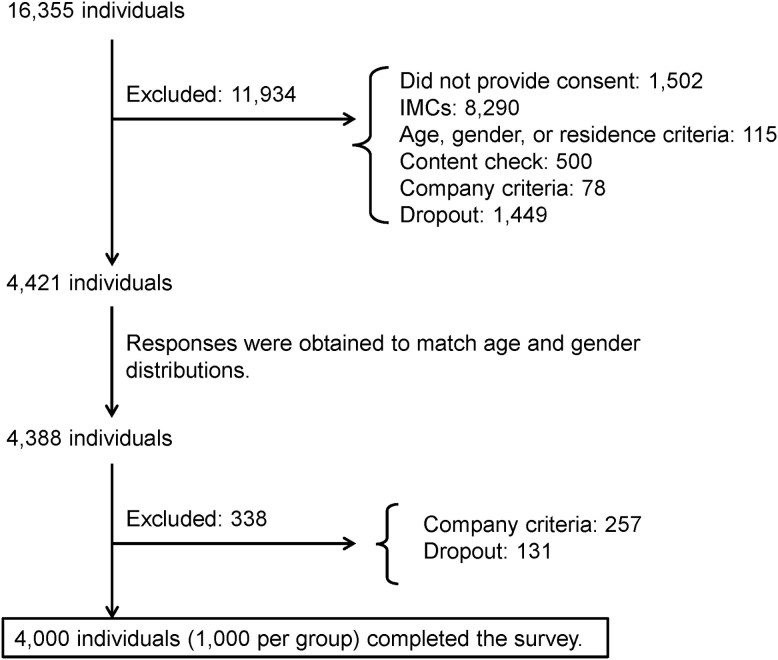
Flow diagram of participant exclusion and selection, showing the reduction from 16,355 respondents to a final sample of 4,000 after applying multiple exclusion criteria. IMCs: incorrect responses to both instructions; age: <20 or >69 years; gender: other; residence: Fukushima Prefecture or outside Japan; content check: incorrect responses to both questions; company criteria: extremely short response time and other quality-control exclusions.

**Table 1:** Demographics, acceptance of final disposal, negative emotions and socio-psychological characteristics by information type. Reliability coefficients: Spearman–Brown (SB) for two-item scales and Cronbach’s α for scales with three or more items

		Mean (standard deviation) or *n (%)*				*r, φ*, or Cramér’s *V*	*P*
		A	B	C	D		
Age		46.1 (13.3)	46.1 (13.2)	46.1 (13.4)	45.9 (13.2)	0.008	.982
Gender	Women	*498 (49.8%)*	*498 (49.8%)*	*498 (49.8%)*	*498 (49.8%)*	<0.001	1.000
	Men	*502 (50.2%)*	*502 (50.2%)*	*502 (50.2%)*	*502 (50.2%)*		
Region	Hokkaido, Tohoku and Kanto	*481 (48.1%)*	*506 (50.6%)*	*502 (50.2%)*	*486 (48.6%)*	0.021	.623
	Chubu, Kinki, Chugoku, Shikoku and Kyushu	*519 (51.9%)*	*494 (49.4%)*	*498 (49.8%)*	*514 (51.4%)*		
Occupation	Employees	*491 (49.1%)*	*476 (47.6%)*	*461 (46.1%)*	*499 (49.9%)*	0.025	.540
	Self-employed	*45 (4.5%)*	*52 (5.2%)*	*60 (6.0%)*	*48 (4.8%)*		
	Other	*464 (46.4%)*	*472 (47.2%)*	*479 (47.9%)*	*453 (45.3%)*		
Marital status	Unmarried	*439 (43.9%)*	*427 (42.7%)*	*410 (41.0%)*	*444 (44.4%)*	0.026	.508
	Married	*506 (50.6%)*	*505 (50.5%)*	*531 (53.1%)*	*489 (48.9%)*		
	Divorced or bereaved	*55 (5.5%)*	*68 (6.8%)*	*59 (5.9%)*	*67 (6.7%)*		
Children	No children	*585 (58.5%)*	*578 (57.8%)*	*561 (56.1%)*	*567 (56.7%)*	0.019	.700
	Have children	*415 (41.5%)*	*422 (42.2%)*	*439 (43.9%)*	*433 (43.3%)*		
Acceptance		2.04 (0.79)^a^	2.11 (0.82)^a^	2.04 (0.83)^a^	2.12 (0.84)^a^	0.051	.030
Negative emotion (involuntariness)		3.54 (1.04)^a^	3.42 (1.03)^b^	3.27 (1.06)^c^	3.42 (1.03)^b^	0.126	<.001
Negative emotion (forced nature)		3.54 (0.99)^a^	3.42 (1.02)^a, b^	3.39 (1.04)^b^	3.49 (1.02)^a, b^	0.069	.006
Negative emotion (distress)		3.20 (1.03)	3.18 (1.09)	3.24 (1.05)	3.18 (1.06)	0.027	.485
Negative emotion (need for improvement)		3.73 (0.90)^a^	3.60 (0.96)^b^	3.71 (0.93)^a, b^	3.70 (0.94)^a, b^	0.065	.016
Inequity (α = 0.84)		3.36 (0.93)	3.28 (0.96)	3.33 (0.95)	3.29 (0.97)	0.043	.199
Intergenerational expectation (α = 0.94)		2.00 (0.87)^b^	2.15 (0.94)^a^	2.09 (0.91)^a, b^	2.05 (0.90)^a, b^	0.079	.004
Social benefits (SB = 0.81)		3.01 (0.96)	3.09 (0.94)	3.05 (0.96)	3.10 (0.95)	0.048	.164
Protected values (SB = 0.96)		3.03 (1.06)^a^	2.93 (1.09)^b^	3.04 (1.10)^a^	2.94 (1.12)^a, b^	0.056	.017
Dread risk perception (α = 0.91)		3.18 (0.78)^a, b^	3.14 (0.78)^b^	3.23 (0.76)^a^	3.17 (0.78)^b^	0.062	.038

^a–c^Different letters indicate significant differences between groups after Bonferroni adjustment (*P* < 0.05).

*r* Effect size between the groups showing the minimum and maximum values.

Contrary to Hypothesis 2, negative emotions differed across information, whilst changes in acceptance and socio-psychological factors did not fully support the other hypotheses. The absence of acceptance differences likely reflects limited influence of information on robust traits such as inequity and social benefits. In contrast, intergenerational expectations, protected values and dread risk perception were affected by information B. Emphasizing Fukushima residents’ burden increased perceived expectations from others and attenuated absolutist rejection and dread risk perception. Although this burden underlies the policy of disposal outside the prefecture [8], highlighting it may foster empathy by encouraging consideration for those most affected by the accident [9], resulting in favourable changes in intergenerational expectations and protected values, as well as reductions in dread risk perception. Information A elicited higher involuntariness, forced nature and need for improvement, suggesting that providing no rationale triggers negative emotions. Overall, because B showed a pattern of higher intergenerational expectations, lower protected values and dread risk perception, greater acceptance and more favourable emotional responses, explaining Fukushima’s burden may be practically beneficial for promoting final disposal strategies [10].

Limitations include potential online participation bias and restriction to individuals aged 20–69, warranting caution regarding generalizability. Furthermore, the effects of information focusing on other factors, including the perception of radiation risks, have not been sufficiently examined. Nevertheless, by clarifying how information influences acceptance and emotional responses, this study provides insights relevant to advancing final disposal.

## Presentation at a conference

No.
